# Epulis of Lower Jaw

**Published:** 1876-07

**Authors:** 


### ARTICLE VII.

### *Epulis of Lower Jaw.*

Dr. Robinson presented a specimen of the above with the following history:—J. P., German women, aged 44 years; does housework; eighteen years in the United States; has had eight children—five living all healthy; she has always been perfectly well; came under my care one month since. About three years ago noticed a tumefaction of the gums at the left of the median line of the lower jaw; the swelling increased very slowly until beginning of last pregnancy—two and one-half years ago—when it took on a more rapid growth. During her period of pregnancy it increased rapidly and reached its present size—that of a cherry. Since birth of last child, nine months ago, it has not augmented. It has rarely been painful and has more particularly caused her mechanical trouble by its presence interfering somewhat with the closing of her jaws, and consequently with mastication. In wet weather she has remarked that the tumor becomes somewhat sensitive.

The tumor presented the aspect of a small globular mass about the size of a cherry. It is tolerably smooth, of a rosy color, very consistent, and not at all pulsating to the touch. It adheres by a broad base or pedicle, the periosteum of the alveolus of the left lateral incisor tooth of the lower jaw. It is not adherent, apparently, to the bony structure of the jaw, and is somewhat movable upon its base. When moved from side to side there is a little bleeding about its base, seemingly brought on by an ulcerative process caused by the neighboring teeth, which it has pushed aside by its own pressure in growing. It is difficult to say positively whether the incisor tooth at the seat of the growth is incorporated with it. The patient thinks so, as she does not remember having lost a tooth at this region. The teeth of the patient are generally defective, and she has already lost several.

About three weeks ago I excised this tumor. At first I attempted to dissect it away from the gums by means of the bistoury. In this I failed, as it was of too hard consistence. I therefore used the bone forceps, cutting down on either side of it, until I had penetrated nearly to the base the alveolus, and then by means of a gouge-shaped dental forceps tearing away tumor with a small portion of bone. By way of precaution against return of growth, I was compelled to extract one incisor tooth and after the operation was terminated and the bleeding arrested I cauterized the base with nitrate of silver.

*Upon section* the tumor presented a tolerable homogeneous structure to the naked eye, and was apparently largely made up of fibrous tissue. There was no tooth enclosed in its tissue. The microscope shows it to be a so called fibro-plastic tumor, or spindle-celled sarcoma.

One of the points of interest in the case had reference to the liability of return. In the instance in question the glands in the neighborhood, as Dr. R. believed was usual in cases of pure sarcomatous tumors, were not involved. He thought from a chemical point of view it was of importance to note the fact of the freedom of glandular infiltration, as bearing upon non-malignancy.—*New York Path. Society.*—*Med. Record.*

